# Prevalence and Factors Associated with Inconsistent Condom Use among Men Who Have Sex with Men (MSM) Who Use Mobile Geo-Social Networking Applications in Greater Tokyo

**DOI:** 10.3390/ijerph15122815

**Published:** 2018-12-10

**Authors:** Adam O Hill, Benjamin R Bavinton, Gregory Armstrong

**Affiliations:** 1 Faculty of Arts and Humanities, University of Melbourne, Parkville, VIC 3010, Australia; 2 Kirby Institute, University of New South Wales, Sydney, NSW 2052, Australia; bbavinton@kirby.unsw.edu.au; 3 Nossal Institute for Global Health, Melbourne School of Population and Global Health, University of Melbourne, Parkville, VIC 3010, Australia; g.armstrong@unimelb.edu.au

**Keywords:** MSM, inconsistent condom use, gay mobile applications, HIV/AIDS prevention

## Abstract

This study examined the prevalence and factors associated with inconsistent condom use among men who have sex with men (MSM) who use gay mobile geo-social networking applications (gay mobile apps) in Greater Tokyo. Among a sample of 1657 MSM recruited through advertisements on gay mobile apps, inconsistent condom use was reported by over one-third (37%) of participants with regular male partners, 18% with casual male partners, and 20% with female partners. In multiple regression analysis, inconsistent condom use with both regular and casual male partners was more commonly reported among participants without a university education, and among participants reporting lower self-efficacy for safer sex. Inconsistent condom use with casual male partners was more commonly reported among participants living in the central 23 wards of Tokyo. Inconsistent condom use with regular male partners was more commonly reported among participants who identified as a member of the gay community, and who only had male partners. These results indicate that a substantial proportion of Greater Tokyo gay mobile app users use condoms inconsistently, particularly with regular partners, and may be at risk for HIV. This paper provides useful information to help design tailored strategies to reduce inconsistent condom use.

## 1. Introduction

MSM (men who have sex with men) are estimated to make up between 2.87% of the male population [1] in Japan, but accounted for 72.7% of HIV cases between 2011–2015 among Japanese nationals [2]. MSM likely account for a greater proportion of HIV and AIDS cases in Japan than surveillance suggests due to underreporting of homosexual transmission [3]. MSM-related HIV surveillance in Japan faces a variety of difficulties including a lack of sustainable financing and limited researchers [4], while MSM uptake of prevention services is hindered by community stigma, Japanese heteronormative cultural values expecting marriage and children, and the limited experience and knowledge of the specific prevention needs facing MSM among health workers [3].

Condoms are one of the main tools for HIV prevention, with a high level of effectiveness at preventing the transmission of HIV and STIs when used correctly and consistently [5]. Despite the wide availability of condoms in Japan, where they are largely available through NGOs (non-governmental organizations) and gay venues for free [6], condom use has remained inconsistent among MSM in Japan [2,7].

Gay geo-social networking applications (hereafter, gay mobile apps) use the global positioning systems (GPS) in smart phones to see other nearby users who they can contact, facilitating MSM meetings [8,9]. Since 2012 gay mobile apps have eclipsed gay bars as a space to meet potential sex partners in Japan [10] and provide access to MSM not accessible through venue-based sampling methods [8,11,12,13,14]. Although there is no research regarding comparing gay mobile app using MSM to non-app using MSM in Japan, previous studies in the U.S. and China have shown gay mobile app users to be at higher risk for STIs [15,16], and to have more sexual partners [9,12,16] when compared to non-app using MSM. Existing research on condom use is largely based on samples of MSM recruited at a range of gay venues [2,4,7], although it has been estimated that only 35% of MSM in Japan attend such gay venues [17]. It is therefore essential to better understand the use of condoms among MSM in Japan who use gay mobile apps. This study seeks to fill this gap, by examining the prevalence and correlates of inconsistent condom use among gay mobile app users in Tokyo, the city at the center of the HIV epidemic in Japan [18].

## 2. Materials and Methods

The survey initially recruited participants using a methodology previously used in the U.S. [8,19] by selecting and messaging nearby users with a link to the survey using the GPS function from 22 November 2016 to 16 January 2017, and recruited 215 valid participants. The gay mobile apps used for recruitment use geo-location to sort users by proximity, with the first profile the closest to the user. Users were displayed in a grid of photos, with three photos per row. The researcher was positioned in Tokyo centrally, launched the applications, and randomly selected one user from each row in a grid of photos until 50 previously uncontacted users were messaged with a link to the survey. Messages were logged in the app chat function, and previously messaged MSM were not contacted again. Questions regarding the survey were also answered via the app chat function. Slow recruitment due to frequent social networking services (SNS) scamming in Japan [20] and potential recruitment bias led to a change in recruitment strategy. A splash screen poster encouraging application users to respond to the linked survey was placed on the most popular gay mobile app in Japan, 9Monsters, for one week from 17 January to 23 January 2017 to supplement response numbers. This methodology was used previously with success in the U.S. [13,14]. The advertisement was displayed only to MSM who used gay mobile apps in Greater Tokyo. It was shown in rotation whenever the application was opened, and could be seen in the ‘advertising’ section in Greater Tokyo, recruiting a further 1442 participants for a total of 1657 valid respondents.

The survey was anonymous, with no identifying information obtained, and was self-administered online. Male participants of 18 years or older, who identified as MSM (defined as identifying as ‘gay’ or ‘bisexual’ or having sexual experience with other men) and gave participation consent were included in results for analysis. The questionnaire was translated from English into Japanese by a native translator and back-translated into English by an independent translator. Duplicate IP addresses and incomplete responses were checked and removed, informed consent was obtained from all survey respondents, and MSM helplines and website information for services was provided to all people who clicked on the survey link. Participants could opt into receiving the survey results by email and could also opt into a lottery for prizes of up to 80 USD in gift cards. In total, 1335 (81%) requested lottery entry, and 964 (58%) requested the survey results. The Human Research Ethics Committee at the University of Melbourne in Australia provided ethics approval (ID: 1646197).

Socio-demographics were measured by 12 items used in prior research in Japan [21], including age, gender, sexual orientation, marital status, birthplace, current residence, self-rated health, education, occupation, work hours, and intercourse partners. Gay mobile app use motivations were defined as ‘to find sex’, ‘to find friends’, ‘to find a serious relationship’, or ‘to avoid being identified as gay’ [22]. Gay community participation measured years and frequency of gay bar and gay event attendance, frequency of gay bathhouse (*hattenba*) attendance, organized gay group activity participation in past six-months, and identity as a gay community member. Four questions about self-efficacy for safer sex were derived from previous instruments [23,24,25], and measured participant confidence in performing specific behaviors with a partner: ‘I feel confident in using condoms even when my partner doesn’t want to’, ‘I am able to avoid behavior that puts me at risk of HIV infection’, ‘I find it difficult to discuss condom use with partners’, and ‘I find it difficult to practice safer sex when I have been drinking’.

Respondents were asked three questions regarding frequency of condom use during penetrative or receptive anal intercourse with regular and casual male partners as well as sex with female partners. Condom use frequency was recorded as ‘never’, ‘rarely’, ‘most of the time’, and ‘always’. Following previous studies, inconsistent condom use was defined as ‘never use condoms’ or ‘rarely use condoms’, while consistent condom use was defined as ‘always use condoms’ and ‘use condoms most of the time’ [26]. Condom use analyses excluded those who did not have intercourse with each respective partner type.

All analysis was performed in SPSS v24 (IBM, Armonk, NY, USA). Descriptive statistics are presented for all variables. Correlates of condom use stratified by intercourse partner type were determined with logistic regression. Unadjusted and adjusted odds ratios (AOR) are presented, along with their 95% confidence intervals (CI). To develop a parsimonious model, only variables with a *p*-value less than 0.10 in univariate analyses were included in the multivariate analyses. Only models with acceptable diagnostics are presented; goodness of fit was assessed using the Hosmer–Lemeshow test, and collinearity was assessed against the variance inflation factor (VIF).

## 3. Results

### 3.1. Socio-Demographic Characteristics

The socio-demographic characteristics of the 1657 MSM using gay mobile apps in Greater Tokyo are displayed in Table 1. Over half (53.4%) resided in central Tokyo, and almost all (96.3%) respondents were born in Japan. The median age was 35, two-thirds (68.5%) were employed fulltime, over half (58.1%) had a university education, and a small minority (4.4%) were married to women. Most participants identified as homosexual (85.1%) and 14.1% as bisexual; 8.9% reported having both male and female sex partners. While the majority of respondents identified as male, 14 participants identified as MSM but were non-binary regarding gender or female-to-male (FTM) transgender. About half (56.8%) of the participants had ever attended a gay bar and half (47.6%) had attended a gay bathhouse (hattenba), with 13.2% having participated in a gay group activity in the past six months.

### 3.2. Condom Use and Self-Efficacy for Practicing Safer Sex Characteristics

Inconsistent condom use was more commonly reported with regular male partners than with casual male partners or female partners (Table 2). Inconsistent condom use was reported by over one-third (37%) of participants with regular male partners, 18% with casual male partners, and 20% with female partners. There was low risk perception among respondents engaging in condomless anal intercourse (CLAI): of 882 respondents who felt that they avoided HIV risk behaviors, over half (53.7%) engaged in CLAI with a regular partner, one-third (33.0%) engaged in CLAI with casual partners, and over one-third (38.5%) engaged in condomless sex with female partners. Of respondents with both male and female partners, 11.3% of participants reported inconsistent condom use with both regular male and female partners, and 8.1% with both casual male and female partners.

Over nine-tenths (91.7%) of participants did not feel they have difficulty discussing condom use with partners, and two-fifths (40.8%) felt confident in their ability to use condoms when a partner does not want to. Just over half (53.9%) of respondents felt they could avoid behavior putting them at risk of HIV infection, and almost half (43.6%) of respondents felt that they had trouble having safe sex when drunk.

### 3.3. Correlates of Inconsistent Condom Use with Regular Male Partners

Multivariate logistic regression analyses found that inconsistent condom use with regular male partners was more frequently reported among participants who were married (AOR, 2.01; 95% CI, 1.09–3.71; Table 3) and who identified as a member of the gay community (AOR, 1.58; 95% CI, 1.13–2.22), and less frequently reported among MSM who: were educated above high school education (e.g., for respondents with a B.A., adjusted odds ratio (AOR), 0.65; 95% CI, 0.48–0.88), had both male and female partners (AOR, 0.58; 95% CI, 0.36–0.93), were students (AOR, 0.51; 95% CI, 0.30–0.86) as compared to those with full-time employment, recently participated in organized gay community activities (AOR, 0.52; 95% CI, 0.35–0.77), were using gay mobile apps in order to make friends (AOR, 0.75; 95% CI, 0.56–1.00), and reported positive self-efficacy for practicing safer sex.

### 3.4. Correlates of Inconsistent Condom Use with Casual Male Partners

Multivariate logistic regression analyses found that the odds of inconsistent condom use with casual male partners were lower among MSM who: were educated above high school education (e.g., for respondents with a BA, AOR, 0.36; 95% CI, 0.24–0.53; Table 4), lived outside central Tokyo (e.g., for a prefecture outside Greater Tokyo, AOR, 0.54; 95% CI, 0.30–0.97), were using gay mobile apps in order to make friends (AOR, 0.65; CI, 0.45–0.94), and had higher self-efficacy for practicing safer sex. The odds of inconsistent condom use with casual male partners were higher among MSM who were using gay mobile apps in order to find sex partners (AOR, 2.08; 95% CI, 1.38–3.14).

### 3.5. Correlates of Inconsistent Condom Use with Female Partners

Multivariate logistic regression analyses found that the odds of inconsistent female partner condom use were lower among MSM who had a two-year university or technical degree (e.g., AOR, 0.75; 95% CI, 0.57–0.99; Table 5), and were higher among MSM who were married (AOR, 2.70; 95% CI, 1.09–6.69).

## 4. Discussion

Condom use is an important determinant of HIV incidence among MSM, and to our knowledge, this is the first study to examine the correlates of inconsistent condom use among gay mobile app users in Japan. Factors associated with inconsistent condom use with regular and casual male partners varied. Inconsistent condom use with casual male partners was more commonly reported among participants without a university education, those living in the central 23 wards of Tokyo, and among participants reporting lower self-efficacy for safer sex. Inconsistent condom use with regular male partners was more commonly reported among participants without a university education, who identified as a member of the gay community, who only had male partners, and among participants reporting lower self-efficacy for safer sex.

Inconsistent condom use was more common than among previous surveys of gay mobile app users outside Japan [27]. Participants reported similar levels of inconsistent condom use with casual male partners, and more frequent inconsistent condom use with regular male partners than previous venue-based studies reporting on condom use consistency in Greater Tokyo [2]. As with previous findings [28,29], condom use was higher with casual male partners (82.6%) and female intercourse partners (80.1%) than regular male partners (63.1%). One-third (31.5%) of respondents reported always using a condom with a regular male partner, lower than neighboring countries such as Cambodia [30], but similar to China [31]. MSM practicing negotiated safety with regular partners (where regular partners agree not to engage in CLAI with outside partners after being tested with regular partners) [32] in HIV negative seroconcordant relationships (where both partners are HIV negative), are associated with low HIV incidence [33]. However, negotiated safety has not been strongly promoted among MSM in Japan. Participants not discussing or complying with negotiated safety is a risk factor for HIV transmission as MSM often were found to have both regular and casual partners concurrently [34], and condom use may become increasingly inconsistent as casual MSM relationships continue [35]. Findings are inconsistent with regards to the associations between regular partner HIV transmissions among MSM, and regular partners were estimated to account for 68% of MSM HIV transmissions in the U.S. [36], but only 10.6% in Australia where negotiated safety has been promoted as a key component of the HIV prevention response among MSM [37]. It is difficult to say to what extent CLAI with regular partners represents a risk unless we know the HIV status, pre-exposure prophylaxis (PrEP) status, and undetectable viral load (UVL) status of the regular partner. Neither Truvada (emtricitabine/tenofovir disoproxil fumarate) (FTC/TDF) nor generic versions of FTC/TDF are approved for prevention in Japan [38]. According to estimates, Japan failed to achieve the first two of the three UNAIDS/WHO (UNAIDS: The Joint United Nations Programme on HIV and AIDS) targets, with 85.6% of HIV-positive cases diagnosed; 82.8% of those diagnosed treated; and 99.1% of those treated experienced viral suppression [39]. U = U (undetectable = untransmissible) messaging was started in Japan in 2018, and had not yet begun at the time of this survey. Regardless, the unavailability of PrEP, and estimated 70.9% of people living with HIV/AIDS (PLWHA) who are undiagnosed or untreated, suggest that MSM engaging in inconsistent consistent condom use with both regular and casual partners may be at risk for HIV in Japan.

While condom use consistency with male partners was comparable to Korea [23], four-fifths of respondents (80.1%) used condoms consistently with female intercourse partners, higher than in MSM in Korea (48.6%; [23]), and China (29%; [40]). This presents less overlap and bridging possibilities for HIV to enter the Japanese heterosexual community through the MSM population, which is important for predicting the long-term pattern of HIV among heterosexuals in Japan [41]. Fewer respondents (40.8%) felt confident in their ability to use condoms when their partner does not want to, compared to 72.3% in Korea [23]. Further studies are needed to determine if this is a specific cultural trait of Japanese MSM or gay mobile app users in general. Moreover, because inconsistent condom use with both regular and casual male partners was reported among participants with low self-efficacy for practicing safer sex, strategies to increase self-efficacy for practicing safer sex such as sexual communication situation-specific rehearsals [42] may improve condom use self-efficacy among MSM in Japan.

Consistent condom use among MSM reporting any anal sex with an HIV-positive partner was found to be 70% effective at preventing HIV infection among MSM [43]. However, condom use among MSM faces various physical challenges, including condom slippage and breakage [44], as well as availability. Condoms are available in all love hotels (short-stay hotels available for sexual activities common throughout Japan), and are provided to gay venues such as bars and gay bathhouses by NGOs in Japan. However, delivery of free condoms to gay mobile app users who do not attend gay venues is problematic. These results identify groups of gay mobile app users that report inconsistent condom use with male partners that can potentially be targeted by interventions. For example, similar to previous findings among MSM in the U.S. [45], education had a protective effect for respondents, and participants with no university education reported three-times the likelihood of inconsistent condom use with casual male partners than university-educated participants, likely due to limited sex education implementation in Japanese junior high and high schools [9]. A trial in Japan using an extended HIV education program in junior high schools has shown potential as an effective HIV prevention tool [46]. In order to reduce inconsistent condom use, sexual minority inclusive sex education programs should be implemented in Japanese middle and high schools.

Moreover, inconsistent condom with casual male partners was more frequently reported among participants living in Central Tokyo than in Greater Tokyo or other prefectures. In order to improve condom use consistency among these groups, in-app promotion of condom use, which has the potential to engage high risk MSM populations [47], could be implemented in central Tokyo in order to reach the large proportion of non-venue attending MSM. Inconsistent condom use was more frequently reported with regular male partners among participants who identified as members of the gay community and who only had male partners, possibly due to more inconsistent condom use with trusted partners [48]. Future condom use interventions targeting gay community MSM should therefore promote the importance of condom use with both regular and causal partners.

Studies show alcohol and drugs to be associated with HIV risk among MSM communities [49]. High rates of binge drinking [11] and alcohol use during sex [8] have been found among gay mobile app users. In this study over three-quarters of survey participants had access to regular drinking partners, and 43.6% of respondents had trouble having safe sex while drunk. Drinking is a common part of Japanese and South-East Asian masculinity and work culture, associated with female sex-worker (FSW) visits [50], and may be associated with HIV risk behaviors with male sex workers (MSW), or with mental stress from heteronormative expectations of FSW visitation and subsequent unwanted ‘outing’. Behavioral interventions that reduce alcohol consumption and increase self-efficacy for safer sex when intoxicated may be important adjunctive approaches in MSM and reduce HIV transmission among MSM in Japan [51].

Evidence shows that daily pre-exposure prophylaxis (PrEP) use reduces risk of getting HIV through sex among HIV-negative people by more than 90% [52]. When considering the levels of inconsistent condom use among Greater Tokyo MSM, legalization and subsidization of PrEP in Japan for high-risk populations such as MSM is likely to help prevent HIV among MSM who engage in CLAI for a variety of individual, structural, and social reasons [53]. Moreover, antiretroviral adherence successfully meets the UNAIDS 2020 target in Japan. Interventions that improve adherence to antiretrovirals in Japan could be used to develop adherence strategies for HIV-negative MSM prescribed PrEP. Frequent HIV testing, condom use with all partner types, and PrEP should all be promoted while appropriately scaling up surveillance before and after prevention strategy implementation among MSM in Greater Tokyo.

### 4.1. Limitations

The results presented were collected from gay mobile apps and, as is common when recruiting hidden populations online, it used a convenience sampling methodology, which was not based on a probability principle. Respondents were recruited through an advertisement, and those who chose to participate may suffer from self-selection bias and be more interested in the future of the gay community in general. It is also not possible to exactly replicate the survey as it would be with a completely randomized methodology. This limits the possibility of these results being used to draw conclusions about the community, or gay mobile SNS users in Greater Tokyo as a whole from this data as it is not a representative sample. Nonetheless, the sample size obtained is substantial and the information provided on the correlates of condom use is informative for targeted interventions. In order to overcome this issue, future longitudinal studies and use of an online-respondent-driven survey method (ORDS) are recommended in order to create a statistically significant population profile. Secondly, the behaviors and attitudes reported may have been subject to social desirability bias where participants may downplay certain behaviors they believe to be undesirable [54]. However, because the survey was anonymous and online we expect that this effect had been minimized. This research only used a selection of popular gay mobile apps, and other gay mobile apps may be associated with different MSM subpopulations important for future research. Other gay mobile apps utilize GPS similarly, and this study method would likely be appropriate for future research in Japan. Lastly, there are only limited validated measures in Japanese available, and future research should also aim to validate scales for use in Japan to collect information on condom use and self-efficacy.

## 5. Conclusions

This research shows that a substantial proportion of Greater Tokyo gay mobile app users use condoms inconsistently, particularly with regular partners. Gay mobile app users are accessible and generally willing to participate in HIV research and prevention via apps. Unlike many gay mobile apps, Japanese app makers have shown willingness to discount prevention and research projects for MSM users. In light of limited MSM research and prevention funding in Japan, and size of the gay mobile app user base, utilization of popular gay apps to promote condom use among all partner types and HIV testing facilities may be an effective prevention policy to target Japanese MSM.

## Figures and Tables

**Table 1:** Socio-demographic characteristics.

		*n*	%
Current residence	Tokyo	883	53.4
	Greater Tokyo	553	33.4
	Another prefecture	209	12.6
	Another country	8	0.5
	Total	1653	100
Birthplace	Japan	1593	96.3
	Other	62	3.7
	Total	1655	100
Age	18–25	319	19.4
	26–35	550	33.4
	36–45	507	30.8
	46+	270	16.4
	Total	1646	100
Gender	Male	1641	99.2
	Other	14	0.8
	Total	1655	100
Marital status	No	1582	95.6
	Yes	72	4.4
	Total	1654	100
Occupation	Full-time	1133	68.5
	Part-time	185	11.2
	Student	168	10.2
	Self-employed	73	4.4
	Freelance	16	1.0
	Unemployed	69	4.2
	Other	9	0.5
	Total	1653	100
Education	High school or less	421	25.4
	Two-year technical school	274	16.5
	University	800	48.3
	M.A.	135	8.2
	PhD	26	1.6
	Total	1656	100
Intercourse partner	Men	1488	91.1
	Men and women	146	8.9
	Total	1634	100
Sexuality	Homosexual	1408	85.1
	Bisexual	233	14.1
	Other	14	0.8
	Total	1655	100
Health	Very unhealthy	17	1.0
	Unhealthy	114	6.9
	Average	467	28.2
	Healthy	816	49.3
	Very healthy	240	14.5
	Total	1654	100.0

**Table 2:** Frequency of condom use and self-efficacy for practicing safer sex.

		*n*	%
How often do you use a condom with a regular (penetrative and receptive anal sex) male partner? ^a^	No anal sex with regular male partner	246	14.9
	Never	217	15.5
	Rarely	302	21.5
	Most of the time	443	31.6
	Always	442	31.5
	Total	1404	100
How often do you use a condom with casual (penetrative and receptive anal sex) male partners? ^b^	No anal sex with casual male partner	318	19.3
	Never	35	2.6
	Rarely	197	14.8
	Most of the time	490	36.8
	Always	609	45.8
	Total	1331	100
How often do you use a condom with female (penetrative and anal sex) partners? ^c^	No sex with female partner	1358	82.6
	Never	29	10.1
	Rarely	28	9.8
	Most of the time	68	23.8
	Always	161	56.3
	Total	286	100
It’s difficult to talk about condom use with partners	Strongly disagree	615	37.6
	Disagree	561	34.3
	Neutral	325	19.9
	Agree	106	6.5
	Strongly agree	30	1.8
	Total	1637	100
I feel confident in my ability to use condoms when my partner doesn’t want to use them	Strongly disagree	103	6.3
	Disagree	283	17.2
	Neutral	588	35.7
	Agree	399	24.3
	Strongly agree	272	16.5
	Total	1645	100
I am able to avoid behavior that may put me at risk of HIV infection	Strongly disagree	48	2.9
	Disagree	153	9.3
	Neutral	554	33.8
	Agree	614	37.5
	Strongly agree	268	16.4
	Total	1637	100
I find it difficult to practice safe sex when drunk	Strongly disagree	202	12.3
	Disagree	274	16.7
	Neutral/disagree	451	27.5
	Agree	556	33.9
	Strongly agree	159	9.7
	Total	1642	100.0

^a^ Analysis based on a subsample of 1404 people who have anal sex with a regular male partner, ^b^ analysis based on a subsample of 1331 people who have anal sex with a casual male partner, ^c^ analysis based on a subsample of 286 people who have sex with women.

**Table 3:** Multivariate binary logistic regression for inconsistent condom use with regular male partners (*n* = 1404).

	Number of Respondents (*n*)	% Reporting Inconsistent Condom Use with Regular Male Partner	Unadjusted Odds Ratio (95% CI)	*p*-Value	Adjusted Odds Ratio (95% CI)	*p*-Value
Age (years)						
18-25	271	31.4	REF		REF	
26–35	482	37.1	1.15 (0.98–1.35)	0.078	1.02 (0.83–1.26)	0.823
36–45	429	37.8	1.11 (1.00–1.23)	0.059	0.96 (0.83–1.11)	0.572
46+	217	42.9	1.14 (1.04–1.25)	0.006	1.05 (0.93–1.18)	0.469
Place of Birth						
Other	58	27.1	REF			
Japan	1345	37.4	1.61 (0.90–2.88)	0.112		
Education						
High School or less	360	43.3	REF		REF	
Two-year university	232	37.1	0.94 (0.86–1.02)	0.130	0.92 (0.84–1.02)	0.101
University	682	35.0	0.71 (0.54–0.92)	0.009	0.64 (0.47–0.88)	0.005
Graduate degree	130	29.2	0.54 (0.35–0.83)	0.005	0.75 (0.46–1.24)	0.270
Employment						
Full-time work	968	38.2	REF		REF	
Part-time work	157	40.1	1.08 (0.77–1.53)	0.653	0.85 (0.56–1.27)	0.428
Student	144	23.6	0.50 (0.33–0.75)	0.001	0.52 (0.30–0.88)	0.016
Self-employed	82	36.6	0.93 (0.58–1.49)	0.766	0.91 (0.54–1.54)	0.729
Unemployed	51	41.2	1.13 (0.64–2.00)	0.675	1.18 (0.62–2.23)	0.612
Current marital status						
Single	1340	36.2	REF		REF	
Married	63	54.0	2.07 (1.24–3.43)	0.005	2.03 (1.10–3.76)	0.024
Current residence						
Central Tokyo	765	37.9	REF			
Greater Tokyo	446	36.1	0.93 (0.73–1.18)	0.534		
Other prefecture	182	35.7	0.91 (0.65–1.28)	0.586		
Intercourse partners						
Only men	1280	38.0	REF		REF	
Men and women	122	27.0	0.61 (0.40–0.92)	0.018	0.54 (0.34–0.88)	0.013
Health						
Fair/Poor health	498	39.8	REF			
Healthy	904	35.4	0.83 (0.66–1.04)	0.106		
Out to close friends						
No	549	36.6	REF			
Yes	838	37.2	1.03 (0.82–1.28)	0.815		
Identify as a member of the gay community						
No	236	32.6	REF		REF	
Yes	1155	37.7	1.25 (0.93–1.68)	0.144	1.50 (1.07–2.10)	0.020
Use gay apps for sex						
No	511	31.9	REF		REF	
Yes	887	39.9	1.42 (1.13–1.78)	0.003	1.15 (0.89–1.50)	0.291
Use gay apps to find friends						
No	339	41.9	REF		REF	
Yes	1053	35.3	0.76 (0.59–0.97)	0.030	0.73 (0.54–0.97)	0.028
Use gay apps to avoid being identified as gay						
No	1268	36.8	REF			
Yes	115	40.0	1.15 (0.78–1.70)	0.490		
Use gay apps to find a serious relationship						
No	631	40.4	REF		REF	
Yes	756	34.0	0.76 (0.61–0.95)	0.014	0.85 (0.66–1.10)	0.218
Ever attended a gay bar						
No	583	37.2	REF			
Yes	817	36.8	0.98 (0.79–1.23)	0.885		
Participation in gay group/community or volunteer activities						
No	1207	38.9	REF		REF	
Yes	194	25.3	0.53 (0.38–0.75)	0.000	0.51 (0.34–0.75)	0.001
Hattenba attendance						
No	716	34.9	REF			
Yes	684	39.0	1.19 (0.96–1.48)	0.111		
Confident in ability to use condoms when partner doesn’t want to						
No	839	49.8	REF		REF	
Yes	558	17.6	0.22 (0.17–0.28)	0.000	0.31 (0.23–0.41)	0.000
Able to avoid behavior that puts me at risk of HIV infection						
No	654	51.4	REF		REF	
Yes	736	24.0	0.30 (0.24–0.38)	0.000	0.54 (0.41–0.71)	0.000
Difficult to talk about condoms with partners						
No	1280	35.0	0.32 (0.22–0.48)	0.000	0.45 (0.29–0.71)	0.001
Yes	112	62.5	REF		REF	
Difficult to have safe sex when drunk						
No	785	32.1	REF		REF	
Yes	609	43.7	0.61 (0.49–0.76)	0.000	0.77 (0.60–0.99)	0.043
Attend regular drinking parties						
No	314	37.9	REF			
Yes	1079	36.7	0.95 (0.73–1.23)	0.699		

**Table 4:** Multivariate binary logistic regression for inconsistent condom use with casual male partners (*n* = 1331).

	Number of Respondents (*n*)	% Reporting Inconsistent Condom Use with Casual Partner	Unadjusted Odds Ratio (95% CI)	*p*-Value	Adjusted Odds Ratio (95% CI)	*p*-Value
Age (years)						
18–25	245	14.3	REF		REF	
26–35	453	15.2	1.06 (0.85–1.32)	0.630	0.97 (0.72–1.30)	0.828
36–45	424	19.3	1.14 (0.99–1.32)	0.071	1.04 (0.85–1.26)	0.717
46+	202	22.8	1.16 (1.03–1.31)	0.015	1.10 (0.93–1.30)	0.252
Place of Birth						
Japan	1273	17.8	REF		REF	
Other	57	8.8	2.30 (0.91–5.82)	0.079	0.76 (0.25–2.27)	0.625
Education						
High School or less	323	29.1	REF		REF	
Two-year university	224	15.6	0.82 (0.74–0.91)	0.000	0.80 (0.70–0.91)	0.001
University	652	14.1	0.40 (0.29–0.55)	0.000	0.35 (0.23–0.52)	0.000
Graduate degree	132	8.3	0.22 (0.11–0.43)	0.000	0.32 (0.15–0.67)	0.002
Employment						
Full-time work	919	16.9	REF		REF	
Part-time work	146	24.0	1.56 (1.03–2.37)	0.037	1.05 (0.62–1.77)	0.856
Student	135	10.4	0.57 (0.32–1.02)	0.059	0.80 (0.36–1.74)	0.565
Self-employed	76	14.5	0.84 (0.43–1.62)	0.597	0.80 (0.37–1.75)	0.582
Unemployed	53	32.1	2.33 (1.28–4.26)	0.006	1.83 (0.86–3.89)	0.118
Current marital status						
Single	1264	17.4	REF			
Married	66	18.2	1.05 (0.56–2.00)	0.871		
Current residence						
Central Tokyo	732	19.7	REF		REF	
Greater Tokyo	430	15.3	0.74 (0.54–1.02)	0.062	0.62 (0.43–0.90)	0.011
Other prefecture	158	13.3	0.62 (0.38–1.02)	0.061	0.57 (0.31–1.03)	0.064
Intercourse partners						
Only men	1212	17.8	REF			
Both men and women	118	13.6	0.72 (0.42–1.26)	0.246		
Health						
Fair/poor health	472	15.1	REF		REF	
Healthy	857	21.6	0.64 (0.48–0.86)	0.003	0.89 (0.63–1.25)	0.491
Out to close friends						
No	519	17.7	REF			
Yes	794	17.6	0.99 (0.74–1.33)	0.965		
Identify as a member of the gay community						
No	217	18.4	REF			
Yes	1101	17.3	0.92 (0.63–1.35)	0.677		
Use gay apps for sex						
No	414	10.6	REF		REF	
Yes	911	20.5	2.17 (1.53–3.09)	0.000	2.01 (1.33–3.03)	0.001
Use gay apps to find friends						
No	340	21.8	REF		REF	
Yes	980	16.0	0.69 (0.50–0.93)	0.017	0.63 (0.44–0.91)	0.013
Use gay apps to avoid being identified as gay						
No	1202	17.6	REF			
Yes	108	18.5	1.07 (0.64–1.77)	0.801		
Use gay apps to find a serious relationship						
No	587	19.5	REF		REF	
Yes	498	15.7	0.76 (0.58–1.02)	0.064	0.95 (0.67–1.34)	0.756
Ever attended a gay bar						
No	530	17.5	REF			
Yes	797	17.3	0.98 (0.74–1.31)	0.913		
Participation in gay group/community or volunteer activities						
No	1139	18.3	REF		REF	
Yes	190	12.1	0.62 (0.39–0.98)	0.040	0.65 (0.37–1.12)	0.117
Hattenba attendance						
No	619	13.6	REF		REF	
Yes	707	20.7	1.66 (1.24–2.22)	0.001	1.10 (0.77–1.58)	0.588
Confident in ability to use condoms when my partner doesn’t want to						
No	817	24.8	REF		REF	
Yes	506	5.1	0.16 (0.11–0.25)	0.000	0.38 (0.23–0.61)	0.000
Able to avoid behavior that puts me at risk of HIV infection						
No	643	31.3	REF		REF	
Yes	673	4.3	.010 (0.07–0.15)	0.000	0.18 (0.11–0.28)	0.000
Difficult to talk about condoms with partners						
Yes	110	33.6	REF		REF	
No	1207	16.1	0.38 (0.25–0.58)	0.000	0.54 (0.33–0.88)	0.014
Difficult to have safe sex when drunk						
No	744	13.0	REF		REF	
Yes	577	23.2	0.50 (0.37 - 0.66)	0.000	0.74 (0.53–1.05)	0.091
Attend regular drinking parties						
No	296	20.9	REF		REF	
Yes	1024	16.5	0.75 (0.54–1.03)	0.077	1.05 (0.71–1.56)	0.814

**Table 5:** Multivariate binary logistic regression for inconsistent condom use with female partners (*n* = 286).

	Number of respondents (*n*)	% Reporting Inconsistent Condom Use with Female Partner	Unadjusted Odds Ratio (95% CI)	*p*-Value	Adjusted Odds Ratio (95% CI)	*p*-Value
Age (years)						
18–25	73	13.7	REF		REF	
26–35	111	10.8	0.89 (0.57–1.39)	0.602	0.65 (0.38–1.11)	0.113
36–45	67	35.8	1.54 (1.16–2.03)	0.002	1.19 (0.84–1.68)	0.328
46+	33	33.3	1.34 (1.05–1.72)	0.019	1.00 (0.72–1.39)	0.986
Place of Birth						
Japan	276	20.3	2.29 (0.28–18.46)	0.436		
Other	10	10.0	REF			
Education						
High School or less	75	29.3	REF		REF	
Two-year university	47	12.8	0.77 (0.60–0.99)	0.039	0.75 (0.57–0.99)	0.046
University	132	18.2	0.54 (0.28–1.04)	0.066	0.51 (0.23–1.13)	0.096
Graduate degree	32	15.6	0.45 (0.15–1.31)	0.141	0.73 (0.20–2.63)	0.633
Employment						
Full-time work	214	22.0	REF		REF	
Part-time work	18	22.2	1.02 (0.32–3.23)	0.980	1.02 (0.27–3.88)	0.971
Student	37	5.4	0.20 (0.05–0.88)	0.032	0.21 (0.04–1.09)	0.063
Self-employed	15	20.0	0.89 (0.24–3.28)	0.859	1.57 (0.37–6.61)	0.540
Unemployed	2	50.0	3.46 (0.21–57.89)	0.373	1.99 (0.06–64.1)	0.699
Current marital status						
Single	244	15.6	REF		REF	
Married	41	46.3	4.68 (2.31–9.47)	0.000	2.70 (1.09–6.69)	0.032
Current residence						
Central Tokyo	131	21.4	REF			
Greater Tokyo	116	15.5	0.68 (0.35–1.30)	0.240		
Other prefecture	39	28.2	1.45 (0.64–3.26)	0.375		
Health						
Healthy	195	21.0	1.23 (0.65–2.34)	0.524		
Fair/Poor health	90	17.8	REF			
Out to close friends						
No	155	17.4	REF			
Yes	129	22.5	1.38 (0.77–2.47)	0.287		
Identify as a member of the LGBT community						
No	76	21.1	REF			
Yes	210	19.5	0.91 (0.48–1.74)	0.775		
Use gay apps for sex						
No	119	20.2	REF			
Yes	165	19.4	0.95 (0.53–1.72)	0.871		
Use gay apps to find friends						
No	77	28.6	REF		REF	
Yes	204	16.7	0.50 (0.27–0.93)	0.028	0.68 (0.33–1.37)	0.276
Use gay apps to avoid being identified as gay						
No	239	20.5	REF			
Yes	41	17.1	0.80 (0.33–1.91)	0.613		
Use gay apps to find a serious relationship						
No	163	22.7	REF			
Yes	119	16.0	0.65 (0.35–1.19)	0.163		
Ever attended a gay bar						
No	179	17.9	REF			
Yes	105	22.9	1.36 (0.75–2.47)	0.310		
Participation in gay group/community or volunteer activities						
No	265	19.6	REF			
Yes	20	20.0	1.02 (0.33–3.19)	0.967		
Hattenba attendance						
No	169	15.4	REF			
Yes	115	26.1	1.94 (1.08–3.50)	0.280		
Confident in ability to use condoms when my partner doesn’t want to						
No	151	26.5	REF		REF	
Yes	135	12.6	0.40 (0.21–0.75)	0.004	0.51 (0.23–1.11)	0.090
Able to avoid behavior that puts me at risk of HIV infection						
No	116	29.3	REF		REF	
Yes	169	13.6	0.38 (0.21–0.69)	0.001	0.53 (0.25–1.13)	0.101
Difficult to talk about condoms with partners						
Yes	19	31.6	REF			
No	265	19.2	0.52 (0.19–1.42)	0.202		
Difficult to have safe sex when drunk						
No	174	19.0	REF			
Yes	111	21.6	0.85 (0.47–1.53)	0.585		
Attend regular drinking parties						
No	60	25.0	REF			
Yes	224	18.8	0.69 (0.35–1.36)	0.285		
